# Comparison of probabilistic choice models in humans

**DOI:** 10.1186/1744-9081-3-20

**Published:** 2007-04-20

**Authors:** Taiki Takahashi, Hidemi Oono, Mark HB Radford

**Affiliations:** 1 Department of Life Sciences, Unit of Cognitive and Behavioral Sciences, School of Arts and Sciences, The University of Tokyo, 21 COE office, 3-8-1 Komaba, Meguro-ku, Tokyo, 153-8902, Japan; 2 Department of Behavioral Science, Faculty of Letters, Hokkaido University, N.10, W.7, Kita-ku, Sapporo, 060-0810, Japan

## Abstract

**Background:**

Probabilistic choice has been attracting attention in psychopharmacology and neuroeconomics. Several parametric models have been proposed for probabilistic choice; entropy model, Prelec's probability weight function, and hyperbola-like probability discounting functions.

**Methods:**

In order to examine (i) fitness of the probabilistic models to behavioral data, (ii) relationships between the parameters and psychological processes, e.g., aversion to possible non-gain in each probabilistic choice and aversion to unpredictability, we estimated the parameters and AICc (Akaike Information Criterion with small sample correction) of the probabilistic choice models by assessing the points of subjective equality at seven probability values (95%–5%). We examined both fitness of the models parametrized by utilizing AICc, and the relationships between the model parameters and equation-free parameter of aversion to possible non-gain.

**Results:**

Our results have shown that (i) the goodness of fitness for group data was [Entropy model>Prelec's function>General hyperbola>Simple hyperbola]; while Prelec's function best fitted individual data, (ii) aversion to possible non-gain and aversion to unpredictability are distinct psychological processes.

**Conclusion:**

Entropy and Prelec models can be utilized in psychopharmacological and neuroeconomic studies of risky decision-making.

## Background

Studies in psychopharmacology, neuroscience, and behavioral economics have revealed that humans and non-human animals discount the value of probabilistic rewards as the receipt becomes more uncertain ("probability discounting", [1-5]). Because pathological gambling and drug misuse are associated with low degree of aversion to uncertainty in probabilistic choice, it is of psychopharmacological interest to examine probabilistic choice models. In neoclassical economic theory, the expected utility theory [6] has been allowed to express subject' risk-attitude (risk-aversion/seeking). In Kahneman and Tversky's prospect theory [7], an extension of expected utility theory models, a subjective value of a probabilistic outcome is expressed as *Af(p)*, where A is a value of a certain reward and f(p) is a some function of *p*, corresponding to subjective probability weight. However, psychological processes underlying discounting uncertain rewards are yet to be investigated. Several important findings on probabilistic choice are observed in behavioral economic, behavioral ecological and psychopharmacological studies. For instance, (i) behavioral economists have demonstrated that in probabilistic choice, small probabilities are overweighted; while large probabilities are underweighted [7-10], (ii) a theoretical framework for intertemporal choice is useful for analyzing probabilistic choice, by replacing delay in intertemporal choice with an average waiting time until winning, i.e. "odds-against", *O *= (1- *probability*)/*probability *[11-13], (iii) people have aversion to loss of information about outcomes of decision under uncertainty (e.g. ambiguity aversion, [14]). Based on these findings, several types of parametric models of probabilistic choice have been proposed in behavioral economics, ecology, psychopharmacology, and neuroeconomics as introduced below. However, to date, comparison of explanatory power of the probabilistic models, and the relationships between parameters in the probabilistic choice models and psychological processes have not extensively investigated.

### Entropy model

A probabilistic choice model based on information theory and psychophysics has been proposed [15]. One reason for the devaluation of uncertain rewards is aversion to loss of information about outcomes. Uncertainty as lack of information in probabilistic choice can be quantified with Shannon entropy (S = -Σ_*i *_*p*_*i *_*log p*_*i*_)_. _It is to be noted that this type of uncertainty is maximal at *p *= 0.5 (not at *p *= 0) and minimal at *p *= 0 or 1. In addition, people overestimate small probability, i.e., subjective probability is expressed as *p*^*a *^(0 <*a *< 1), following a psychophysical law [15]. Combining aversion to lack of information and overestimation of probability, an impact of probability on a subjective value of an uncertain outcome is *p*^*a*^*- TS *(*a *and *T *are free parameters). Hence, a subjective value *V *of probabilistic reward is:

*V *= *A *[*p*^*a *^- *T*(- [*p log*_2 _*p *+ (1 - *p*)*log*_2_(1 - *p*)])]

where *A *is the value of a certain reward when *p *= 1, 0 <*a <*1 indicates a psychophysical effect on small probability estimation (importantly, smaller *a *values correspond to strong overestimation of small probability, because 0 <*p *< 1) and *T *is a degree of aversion to lack of information [15]. A recent neuroimaging study employed a psychologically similar probabilistic choice model and reported that during anticipation periods in probabilistic choice, degrees of unpredictability were correlated with the activations in an area extending posterior to and bilateral from the ventral striatum to the subthalamic nucleus as well as mediodorsal thalamic nucleus, midbrain, and bilateral anterior insula [16]. The entropy-based model of probabilistic choice has an advantage that each parameter has specific psychological correlates (i.e. psychophysics of probability estimation and aversion to lack of information).

### Prelec's probability weight function

In order to explain subjects' tendency in probabilistic choice; namely, overweighting of small probabilities and underweighting large probabilities, the behavioral economist Prelec has axiomatically derived the following model of probabilistic choice [8]:

*V *= *Aexp *[-*β *(-*ln p*)^α^],

where *α *and *β *are free parameters and *ln *indicates a natural log (= *log*_*e*_). An important characteristic of Prelec's weight function is that this captures human bias in probabilistic choice (i.e. overweighting of small probabilities and underweighting of large probabilities). Notably, when *β *is assumed to be 1, *V *is equal to a statistical expected value = *Ap *at *p *= 1/*e *≈ 0.37, which is reportedly consistent with several behavioral data [8-10]. A recent neuroeconomic study (employing Prelec's weight function with the assumption of *β *= 1, following Prelec's proposal) observed that *α *is related to the activity of the anterior cingulate in the brain, and the authors interpreted the activity of the anterior cingulated might reflect subject's risk attitude in probabilistic choice [17]. However, it is still unknown whether the parameters in Prelec's weight function are actually related to subject's risk aversion (i.e., aversion to possible non-gain in each probabilistic choice and delay until winning, [11]). To examine this question is a part of the objectives of the present study.

### Hyperbolic models

The behavioral psychologists Rachlin et al. hypothesized that a small probability of receipt corresponds to a large delay, based on the molar approach to the concept of probabilistic choice [11]. According to the hypothesis, subjects may discount probabilistic rewards as "odds against", *O *= (1-*p*)/*p *(*p *= probability of receipt in each probabilistic choice), increases (it should be noticed that a larger odds against corresponds to a smaller probability of receipt). Therefore, the "odds against" in probability discounting has been hypothesized to play the same role as the delay D in delay discounting functions in intertemporal choice [11]. Because a general type of delay discounting functions is expressed as the general hyperbolic function, according to Rachlin's hypothesis, a general hyperbolic probability discounting function [18] should be:

*V *= *A*/(1 + *kO*)^*s*^

where *O *= (1 - *p*)/*p *(odds against) and *k *and *s *are free parameters indicating the subject's aversion to possible delay (or possible non-gain in each probabilistic choice). Several authors utilized a simpler form of hyperbolic probability discounting function by setting *s *= 1 (simple hyperbolic function, [11]):

*V *= A/(1 + *kO*).

Psychopharmacological studies have employed these hyperbolic models to describe subjects' probabilistic choice [1-4,19]. However, recent studies have cast a doubt on the hypothetical equivalence of a decrease in probability to an increase in delay; specifically, several studies reported that parameters in delay and probability discounting functions are not so strongly correlated as originally supposed [13,20], and some studies have reported that neurobiological/psychopharmacological manipulation distinctly impacts delay and probability discounting [3,21]. Additionally, a recent study by Bickel's group has proposed an analytical strategy combining delay and probability [22].

In the present study, the goodness of fitness of each equation to the behavioral data in probabilistic choice was quantified with AICc (Akaike Information Criterion with small sample correction, a second-order AIC, [23]) and compared at both group and individual levels. Furthermore, to avoid equation type-dependent systematic errors, we also computed each subject's AUC (i.e., area under the normalized indifference curve) [24] in order to quantify the subject's degree of risk aversion (aversion to possible non-gain in each probabilistic choice). Finally, we computed correlation coefficients between the parameters in probabilistic choice models and individuals' degrees of discounting probabilistic rewards, in order to elucidate a psychological correlate of the parameters of the probabilistic choice models.

## Methods

### Participants

Twenty-one (9 male and 12 female) volunteer students from a major national university in Japan participated in the experiment. The average age was 22.05 (*SD *= 2.2) years. They were recruited from several psychology classes. They participated in a probabilistic choice task, for their parameters of probabilistic choice models to be estimated.

### Procedure

We used exactly the same experimental procedure as Ohmura et al's [20] in the probabilistic choice task. Firstly, participants were seated individually in a quiet room, and face the experimenter across a table. After that, participants received the simple instruction that the monetary reward in this experiment was hypothetical, but the experimenter wanted you to think as though it were real money. Then the participants were asked to choose between the card describing money delivered certainly and the card describing money delivered with a certain degree of probability. The left card viewed from participants indicated the amounts of money that could be received certainly, and the right card indicated 100,000 yen that could be received with a certain probability.

For the probabilistic choice task, monetary rewards and the probability were printed on 3 × 5 index cards. The 27 monetary reward amounts were 100,000 yen (about $1,000), 99,000 yen, 96,000 yen, 92,000 yen, 85,000 yen, 80,000 yen, 75,000 yen, 70,000 yen, 65,000 yen, 60,000 yen, 55,000 yen, 50,000 yen, 45,000 yen, 40,000 yen, 35,000 yen, 30,000 yen, 25,000 yen, 20,000 yen, 15,000 yen, 10,000 yen, 8,000 yen, 6,000 yen, 4,000 yen, 2,000 yen, 1,000 yen, 500 yen, and 100 yen. The seven probabilities of receipt were 95%, 90%, 70%, 50%, 30%, 10%, and 5%.

The experimenter turned the 27 100% cards sequentially. The card started with 100,000 yen, down to 100 yen, and back to 100,000 yen. For each card, the participant pointed the certain (100%) or probabilistic reward. The experimenter wrote down the last 100% reward chosen in the descending order, and the first 100% reward chosen in the ascending order, and the average of them was used as the point of subjective equality (hereafter *indifference point*) in the following analysis. This procedure was repeated at each of the seven probabilities (for more details, [20]).

### Data analysis

We employed both equation type-dependent and -independent parameters. For estimating equation type-dependent parameters (i.e. *a *and *T *in the entropy model, *α *and *β *in Prelec's weight function, *k *and *s *in the general hyperbolic function, and *k *in the simple hyperbolic function). We fitted the four types of the model equations (i.e. Equation 1–4) to the data (R statistical language, non-linear modeling package) and the fitness of each equation was estimated with AICc (Akaike Information Criterion with small sample correction) values, which is the most standard criterion for the fitness of mathematical model to observed data with a small sample size [23]. It should be noted that the comparison between the R-square values of equations with different numbers of free parameters are statistically irrelevant (note that an increase in the numbers of free parameters in a fitting equation always yield a larger R-square value[25]). Therefore, we compared AICc values for the equations (note that smaller AICc values correspond to better fitting). Furthermore, calculating R-square values demonstrated that, the models with two parameters (i.e. Entropy, Prelec, and general hyperbola) had larger R-squre values than the simple hyperbola (which is a statistically trivial result), but no statistically significant difference was observed between the models with two parameters. Because utilizing R-square in non-linear curve fitting is statistically problematic [23], we do not present R-square values below.

For an equation type-independent parameter of probabilistic choice, we adopted AUC, which is defined as the normalized area under the linkage of indifference points (i.e., subjective values) at each odds against (= (1- *probabilit*y)/*probability *[11]). The rationale for employing AUC is that (i) AUC indicates subject's aversion to possible non-gain (or possible temporal delay until winning [11], (ii) AUC does not depend on the type of fitting functions, and (iii) studies in psychopharmacology often utilize AUC as an equation-free parameter for probabilistic choice [3,24].

In order to examine the relationship between the parameters of the probabilistic choice models and subject's degree of aversion to possible non-gain (risk aversion), we utilized Pearson's correlation analyses between the parameters of the model equations and AUC, because Kolmogorov-Smirnov tests revealed no significant deviation from Gaussian distribution in all parameter distributions (*p *> .05). It is to be noted that a significant correlation in the Pearson's analysis indicates that the parameter is related to psychological processes of aversion to possible non-gain (or possible waiting time until winning).

For the analysis of group data, we utilized group median data [26]. The reason is that a (linearly averaged) group mean may not be a valid statistical summary of group data when the relationship between probability values and subjective values is non-linear.

All statistical procedures were conducted with R statistical language (The R Project for Statistical Computing). Data are expressed as Mean and/or Median ± Standard Error of Mean (SEM). Significance level was set at 5% throughout (for pairwise multiple comparisons, Bonferoni's correction was utilized).

## Results

### Fitness of probabilistic choice models for group data

**Figure 1:**
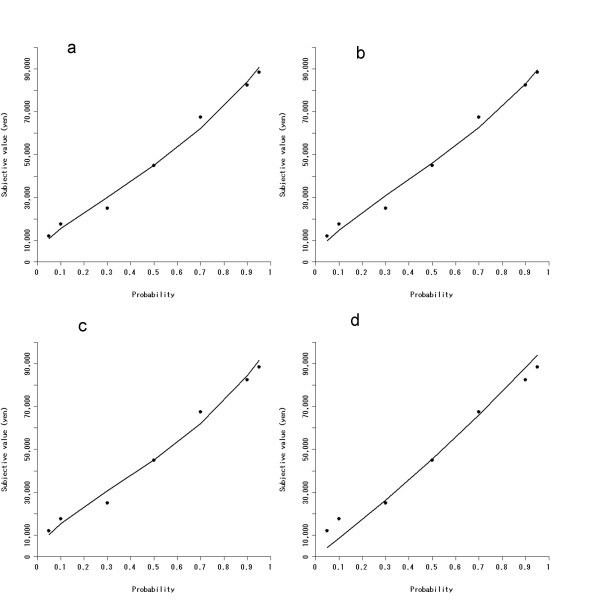
Group median data of probabilistic choice (dots) are presented with lines of prediction from the four types of probabilistic choice models: (a) entropy model, (b) Prelec's weight function, (c) general hyperbolic function, (d) simple hyperbolic function. Horizontal axis indicates probability values (0–1), while vertical axis indicates a subjective value (0–100,000 yen) of the uncertain reward at each value of probability. It can be seen that the simple hyperbolic function poorly fit the data at small and large probability values.

After fitting each model to the group median data, we then employed AICc as an index of fitness (see Table 1 for estimated parameters and AICcs for the group data). As shown in Table 1, *β *in Prelec's weight function was close to 1 as previously reported [8] and general hyperbolic *s *was smaller than 1 as originally proposed [18,25]. The orders of the AICcs for medians were [entropy model < Prelec's weight function < general hyperbolic function < simple hyperbolic function]. Plots between the observed behavioral data and prediction of each model for comparison are presented in Fig. 1. As can be seen, the simple hyperbolic function failed to predict subjective values of the probabilistic reward at small and large probability values, in comparison to the three other probabilistic choice models, indicating that subjects' probabilistic choice has anomalies at small and large probability values (i.e., subjects overweight small probabilities but underweight large probabilities), which cannot exactly be modeled by the simple hyperbolic function.

**Table 1:** Parameters and AICc (Akaike Information Criterion with small sample correction) of probabilistic choice models for group data

	Entropy model		Prelec's function		General hyperbola		Simple hyperbola
AICc	137.96		138.61		139.46		143.05

Parameter	*a*	*T*	*α*	*β*	*k*	*s*	*k*
	
	0.59437	0.21222	0.75456	1.02024	3.21403	0.55207	1.195

For group median data (N = 21) for each indifference point, goodness of fitting was [Entropy model>Prelec's function>General hyperbola>Simple hyperbola]. Note that smaller AIC (Akaike Information Criterion) corresponds to better fitting to behavioral data. It was confirmed that *β *≈ 1, and *s *< 1 for group data.

### Fitness of probabilistic choice models for individual data

Nonlinear curve fitting of the general hyperbolic function to one subject's behavioral data did not converge and this subject's behavioral data were not included in further analysis. Therefore, a total of 20 subjects' data were fitted by each probabilistic choice model. It is important to note that averaged *β *in Prelec's function was close to 1 and *s *in the general hyperbolic function was smaller than 1 as we have shown for group data. Further, corresponding AICcs were calculated (Table 2). A one-way repeated measure ANOVA with equation type (Equation 1–4) as a four-level within-subjects factor revealed a significant difference regarding probabilistic choice models, *F*(3, 19) = 7.5679, *p *< .01. The subsequent *posthoc *pairwise multiple *t*-tests with Bonferoni's correction revealed that Prelec's weighting function significantly better fitted the observed individual data than the simple hyperbolic function (*p *< .05), again indicating that the simple hyperbolic function most poorly fitted the behavioral data. It might be concluded that Prelec's weight function best fitted the individual behavioral data. The estimated individual parameters are summarized in Table 2.

**Table 2:** AICc (Akaike Information Criterion with small sample correction) and parameters of probabilistic choice models for individual data

	Entropy model		Prelec's function		General hyperbola		Simple hyperbola
AIC_c_	133.31 ± 1.79		129.38 ± 1.80		130.82 ± 1.80		139.91 ± 1.80

Parameter	*a*	*T*	*α*	*β*	*k*	*s*	*k*
	
	0.691 ± 0.090	0.197 ± 0.045	0.774 ± 0.068	1.063 ± 0.075	10.910 ± 3.23	0.6348 ± 0.110	1.59 ± 0.33

All values are expressed as mean ± SEM. Note that *β *≈ 1, and *s *< 1 for averaged values, and Prelec's model significantly better fit individual data than Simple hyperbola (p < 0.01, N = 20)

### Correlation between probabilistic model parameters and AUC for individual data

**Figure 2:**
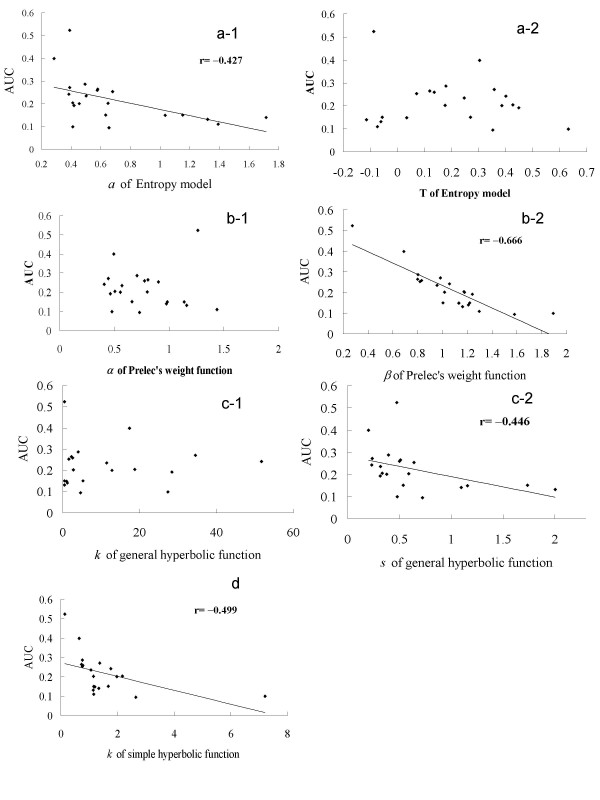
Scatterplots of individual parameter values of each probabilistic choice model (horizontal axis): (a) parameters of entropy model, (b) parameters of Prelec's weight functions, (c) parameters of general hyperbolic function, and (d) a parameter of simple hyperbolic function) and AUC (Area Under the Curve, vertical axis). Note that small AUC indicates subject's strong aversion to possible non-gain in each probabilistic choice (risk aversion). Note that *a *of entropy model, *β *of Prelec's weight function, and *s *of general hyperbolic function, and *k *of simple hyperbolic function were significantly negatively correlated with AUC, while other parameters such as *T *in the entropy model (an indicator of aversion to unpredictability) were not significantly correlated with AUC.

Finally, we examined the correlation between the individuals' parameters for each probabilistic choice model and AUCs (Fig. 2). Among the model parameters, *a *in the entropy model, *β *in Prelec's weight function, general hyperbolic *s*, simple hyperbolic *k *are significantly negatively correlated with AUC (note that smaller AUC corresponds to greater degree of aversion to possible non-gain) (*p *< .05), indicating that these parameters are related to subject's degree of risk aversion. In contrast, *T *in the entropy model (an indicator of subject's degree of aversion to unpredictability) did not significantly correlate with AUC (*p *> .05), implying that risk aversion and aversion to lack of information are distinct psychological processes. It should further be noticed that *α *in Prelec's weight function did not significantly correlate with AUC, implying that *α *may not be an indicator of risk aversion.

## Discussion

To our knowledge, this study is the first to systematically examine the fitness of probabilistic choice models by employing AICc as an indicator of the fitness. Previous studies have focused on the comparison of R-square values; however, this is problematic and statistically irrelevant, because the model equations have different numbers of free parameters and non-linear [18,23,25].

### Comparison of probabilistic choice models at the group level

The order of fitness of the model is [entropy model > Prelec's weight function > general hyperbolic function > simple hyperbolic function]. Although the differences are not dramatically large in a quantitative sense, it may be recommended, in future psychopharmacological studies on probabilistic choice, to utilize the entropy model for examining group differences, in order to elucidate the distinctions in neuropharmacological mechanisms between healthy controls and addicts. Moreover, it should be noted that *β *was close to 1, which confirms that people's probability estimation is the most accurate at the probability value of about 0.37, as Prelec originally proposed and observed in behavioral data [8]. However, this does not necessarily allow us to assume *β *= 1 *a priori*.

### Comparison of probabilistic choice models at the individual level

Prelec's weight function may be the best to parametrize each individual's probabilistic choice, when the objective of the study is to solely reveal individual differences in probabilistic choice. Several authors in neuroeconomics have assumed *β *= 1 and estimate *α *in order to assess individual differences in risk aversion. However, the problem of Prelec's function is that the parameters *α *and *β *do not have clear interpretation, in terms of psychological functioning. We therefore examined the relationships between parameters in each probabilistic choice model and AUC (an index of risk-aversion [11]), in order to better understand the psychological interpretation of parameters in the probabilistic choice models.

### Relationships between probabilistic model parameters and AUC

Our correlational analysis demonstrated that a in the entropy model, *β *in Prelec's weight function, and general hyperbolic *s*, and simple hyperbolic *k *were associated with each subject's risk aversion (assessed with AUC, see Fig. 2). It is important to note that *T *in the entropy model did not relate to risk aversion, confirming that aversion to lack of information and risk are distinct psychological processes [14]. Psychological interpretation for this distinction is that unpredictability-aversive subjects do not necessarily avoid uncertain rewards with very small probabilities (i.e., predictable non-gains), due to predictability of the outcomes. Our result is, as far as we know, the first to dissociate these two attitudes toward uncertainty in a parametric manner. Furthermore, the finding that not *α *but *β *in Prelec's weight function was associated with risk aversion is also important. This finding implies that when the objective of the study is to assess each subject's risk aversion, *β *should not be assumed to be 1. It is also confirmed that simple hyperbolic *k*, rather than general hyperbolic *k *is related to subject's risk aversion. Moreover, as can be seen from Fig. 2, simple hyperbolic parameters at large values are not good predictors of AUC. This is also an important point of cautions for psychopharmacological study on probabilistic choice employing hyperbolic function parameters.

### Limitations and future directions

We now discuss limitations of the present study. Because the present study employed hypothetical money, although discounting behaviors of real and hypothetical rewards have been shown to correlate [27,28], it is not completely evident that the degrees of discounting real monetary gains were exactly reflected. However, probabilistic choice tasks with real money have other limitations; e.g., it is virtually impossible or difficult to utilize large real rewards and real monetary loss, and paying real money for randomly selected participants introduces another probabilistic factor. Nevertheless, real gambling tasks such as the IOWA gambling task [29] may also help understand neuropsychological functioning underlying probabilistic choice. Furthermore, it is also of interest to examine the relationship between ambiguity aversion in decision under uncertainty with unknown probability [14] and subjects' degree of unpredictability aversion, to elucidate substance abusers' risk-taking, and psychopharmacological treatment for impulsive behavior.

## Conclusion

Our present study demonstrated that (i) the entropy model best fit group level data of probabilistic choice, (ii) Prelec's weight function best fit individual data, and (iii) aversions to risk and lack of information are distinct psychological processes. Further studies are required in order to answer whether psychiatrics such as drug-dependent subjects and pathological gamblers actually differ in parameters in the entropy model and Prelec's weight function.

## Competing interests

The authors certify that the information listed above is complete to the best of our original research. The authors declare that they have no competing interests.

## Authors' contributions

TT is the principal researcher of the present study. HO and MHBR also contributed to data collection and experimental design. All authors read and approved the final manuscript.
